# Beta-band frequency shifts signal decisions in human prefrontal cortex

**DOI:** 10.1016/j.isci.2025.113806

**Published:** 2025-10-17

**Authors:** Elie Rassi, Julio Rodriguez-Larios, Camille Gret, Hugo Merchant, Alma Elshafei, Saskia Haegens

**Affiliations:** 1 Donders Institute for Brain, Cognition, and Behaviour, Radboud University, Nijmegen, the Netherlands; 2 Department of Psychology and Centre for Cognitive Neuroscience, University of Salzburg, Salzburg, Austria; 3 Brunel University of London, London, UK; 4 Instituto de Neurobiología, UNAM, Campus Juriquilla, Queretaro, Mexico; 5 Department of Psychiatry, Columbia University, New York, NY, USA; 6 Division of Systems Neuroscience, New York State Psychiatric Institute, New York, NY, USA

**Keywords:** Natural sciences, Biological sciences, Neuroscience, Clinical neuroscience

## Abstract

Beta-band synchronization has been found to be content-specific, particularly during decision-making. Recently, we showed a beta-band frequency shift in macaque prefrontal cortex to reflect categorical decision outcomes in a temporal categorization task. Here, we sought to replicate these findings in human participants, and additionally asked whether these beta frequency shift dynamics generalize to other task contexts and modalities. Across three EEG and MEG (magnetoencephalography) experiments (*n* = 82; 42 female and 40 male) that required participants to make a perceptual decision, we found that a beta-band frequency shift in the prefrontal cortex signaled the decision outcome. This finding was consistent across a temporal categorization task, a delayed match-to-sample task, and a cross-modal discrimination task. We conclude that this signal is a robust marker of perceptual decisions.

## Highlights

• A beta-band frequency shift signals the categorical decision outcome in a time judgment task
• The link between beta-band frequency shifts and decisions generalizes to other 2AFC tasks
• The frequency shift is localized in, but not restricted to the prefrontal cortex
• We replicate previous findings in non-human primates and expand on the role of the beta rhythm

## Graphical abstract

**Figure:**
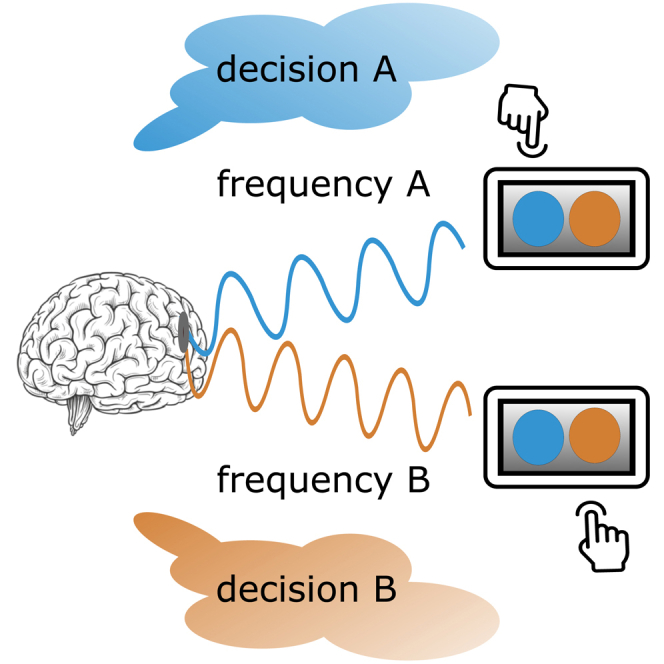

## Introduction

Rhythmic neural activity in the beta frequency range (13–35 Hz) is traditionally associated with sensorimotor processes^1^ such as movement preparation^2^ and motor control.^3^^,^^4^ Beta is additionally being increasingly linked with cognitive functions such as working memory^5^^,^^6^^,^^7^ and decision-making.^8^^,^^9^ Some theoretical accounts have posited that beta-band activity is involved in the maintenance of sensorimotor and cognitive states via top-down mechanisms,^10^ as well as the reactivation of cognitive states or contents.^11^ In this view, a decision can be seen as the reactivation of one of several cognitive states representing decision alternatives.^9^^,^^11^ Beta rhythms could therefore be leveraged as signals of decision outcomes, particularly in the prefrontal cortex, which is thought to carry decision signals.^12^^,^^13^^,^^14^^,^^15^ It remains unknown, however, by which mechanism beta dynamics would signal decisions, with candidates including changes in power, frequency, or connectivity.^16^

**Figure 1:**
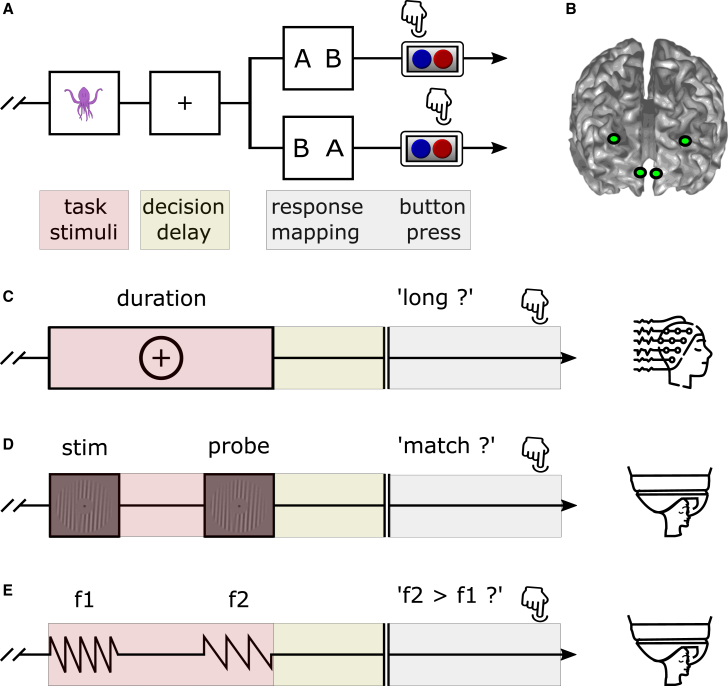
Trial sequences and regions of interest (A) Stimulus presentation was followed by a decision delay in which participants made a decision between two alternatives, but could not yet indicate their decision via button press. Only after the 1.5-s to 2-s decision delay did a response mapping screen indicate to participants which button corresponded to which decision. The response mapping was randomized on a trial-by-trial basis. (B) Regions of interest were left and right dlPFC and left and right vmPFC. (C) In the temporal categorization task (EEG), participants had to categorize the duration of a time interval as being relatively “long” or “short”. (D) In the delayed match-to-sample task (MEG), participants had to indicate whether a probe stimulus was of the same or different spatial frequency as a previously presented stimulus (“match” or “mismatch”). (E) In the cross-modal discrimination task (MEG), participants had to indicate whether the second of two sequentially presented stimuli vibrated at a higher or lower frequency than the first (“faster” or “slower”).

Due to beta’s involvement in motor processes, and because decisions in experiments often result in movements (e.g., button presses), it can be difficult to isolate cognitive (vs. motor) beta rhythms in electrophysiological data. In fact, studies linking beta rhythms to decisions often *a priori* operationalize beta activity as a response-related signal,^17^^,^^18^^,^^19^ potentially obscuring decision-related activity that might occur independently of the subsequent movements. One effective way to disentangle decision-related neural activity from movement-related activity is by introducing a decision delay in task designs, after which a response prompt randomly maps decisions to motor responses^20^ (Figure 1A). By randomizing the response mapping on a trial-by-trial basis and prompting after a decision has been made, one can ensure that a readout of the decision is independent of the subsequent motor output.

Decisions in daily life and in laboratory experiments vary greatly in their scope and depend on context and task demands. This variance makes it difficult to uncover decision signals that generalize across contexts. We tackled this problem in a recent study and found that in macaque prefrontal cortex, a shift in beta frequency signaled context-dependent categorical decisions.^9^ In multiple versions of duration- and distance-categorization tasks,^20^^,^^21^ we changed the boundary between categories, and found that two distinct beta-band frequencies were consistently associated with decisions about the two relative categories, regardless of the objective magnitudes of the stimuli. The first goal of the current study was to test whether a beta-band frequency shift also signals context-dependent categorical decisions in the human prefrontal cortex, using the same task.

Ultimately, we are interested in decision signals that generalize to different tasks that require different kinds of decisions. Further, we want to know if such signals are present regardless of the modality of task stimuli. To these ends, we ran three experiments (total *n* = 82) that each required qualitatively different decisions to be made, but all included a decision delay that was dissociated from the subsequent motor response. In Experiment 1 (EEG [electroencephalography], *n* = 24), we used a context-dependent time-categorization task, where participants decided whether a time interval was relatively “long” or “short.” In Experiment 2 (MEG [magnetoencephalography], *n* = 28), we used a delayed match-to-sample task, where participants decided whether a visual probe stimulus was a “match” or “mismatch” with a previously presented stimulus. In Experiment 3 (MEG, *n* = 30), we used an audio-tactile frequency discrimination task, where participants decided whether a stimulus vibrated at a “higher” or “lower” rate than a previously presented stimulus. In all three experiments, we found that a shift in beta frequency in prefrontal cortex signaled the decision outcome.

## Results

We analyzed data from three experiments and 82 participants in total. On each trial of each experiment, there were two possible choices that participants could make, one of which was correct. Within each experiment, each of the two choices was correct on half of the trials. In Experiment 1 (temporal categorization; EEG; 336 trials across 3 blocks; Figure 1C), the choice was “short” vs. “long,” and on average participants performed with 72.2% (std = 9.1%) accuracy. In Experiment 2 (delayed match-to-sample; MEG; 256 trials across 8 blocks; Figure 1D), the choice was “match” vs. “mismatch,” and on average participants performed with 80.2% accuracy (std = 10.0%). In Experiment 3 (cross-modal discrimination; MEG; 320 trials across 8 blocks; Figure 1E), the choice was “faster” vs. “slower,” and on average participants performed with 73.7% accuracy (std = 7.0%). All three experiments had a decision-making component, where participants were forced to choose between two alternatives after a fixed decision delay. A response map, randomized on a trial-by-trial basis, appeared after the decision delay, prompting participants to respond by pressing one of two buttons (Figure 1A).

### Prefrontal beta power is modulated during the decision delay

Based on the prefrontal cortex’s well-documented involvement in decision-making,^12^^,^^13^^,^^14^^,^^15^ we selected right and left ventromedial (vmPFC) and dorsolateral (dlPFC) prefrontal cortices as our four atlas-defined regions of interest (Figure 1B). We reconstructed source activity at these locations with beamforming techniques. All analyses reported here were performed on these source-level data, unless otherwise specified.

**Figure 2:**
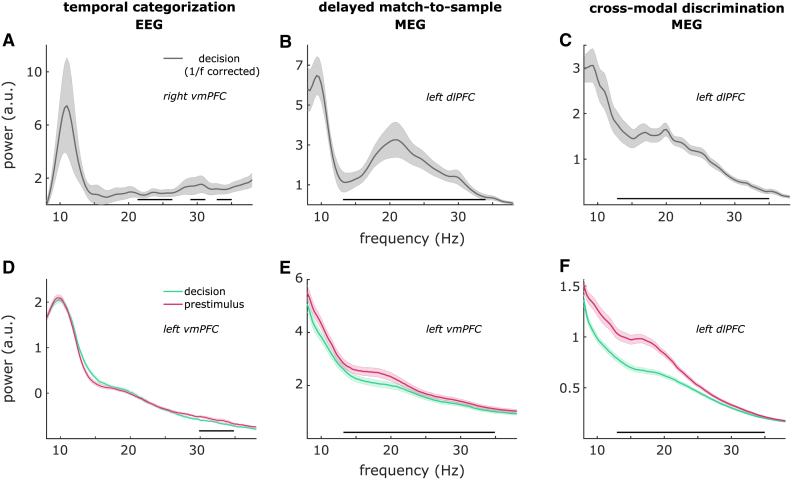
Beta-band power was higher than 1/f background, and suppressed during decision delay (A) 1/f corrected EEG beta power in right vmPFC during decision delay. (B) 1/f corrected MEG beta power in left dlPFC. (C) 1/f corrected MEG beta power in left dlPFC. (D) EEG beta suppression in left vmPFC during decision delay vs. pre-stimulus baseline. (E) MEG beta suppression in left vmPFC. (F) MEG beta suppression in left dlPFC. Shaded regions around the line graphs represent the standard error of the mean. Horizontal black lines above the *x* axis represent the clusters driving the significant differences.

First, to check whether beta power was significantly elevated above what would be expected from 1/f background signal, we estimated the fractal and oscillatory components of the spectral signal and found that in all three experiments, during both the decision delay and a pre-stimulus baseline, oscillatory beta-band power in the prefrontal cortex was significantly increased in all four regions of interest compared to fractal beta-band power (Figure 2). In Experiment 1 (temporal categorization), the increase was most pronounced in left vmPFC in the frequency range of 22.3–27.3 Hz during the pre-stimulus delay (*p* < e−5), and in right vmPFC in the frequency range of 21.3–27 Hz during the decision delay (*p* < e−5; Figure 2A). In Experiment 2 (delayed match-to-sample), the increase was most pronounced in left vmPFC in the frequency range 15.3–32.3 Hz during the pre-stimulus delay (*p* < e−5), and in left dlPFC in the frequency range 13–34.3 Hz during the decision delay (*p* < e−5; Figure 2B). In Experiment 3 (cross-modal discrimination), the increase was most pronounced in left dlPFC and left vmPFC in the entire tested frequency range of 13–35 Hz during both the pre-stimulus delay (*p* < e−5) and the decision delay (*p* < e−5; Figure 2C).

In all three experiments, we found that beta-band power in the prefrontal cortex was suppressed during the decision delay compared to a pre-stimulus baseline (Figure 2), in line with previous reports (e.g.,^6^^,^^9^ in Experiment 1 [temporal categorization], the beta suppression was most pronounced in the left vmPFC in the frequency range of 30–35 Hz [*p*_*cluster*_ = 0.016; Figure 2D]. In Experiment 2 [delayed match-to-sample], the beta suppression was most pronounced in the left vmPFC in the entire tested frequency range of 13–35 Hz [*p*_*cluster*_ < 10e^−4^; Figure 2E]. In Experiment 3 [cross-modal discrimination], the beta suppression was most pronounced in the left dlPFC in the entire tested frequency range of 13–35 Hz [*p*_*cluster*_ < 10e^−4^; Figure 2F]).

In summary, beta power was suppressed in all three experiments during the decision delay compared to baseline, and this suppression was most prominent in the left prefrontal cortex. These results suggest that prefrontal beta rhythms are involved in the decision-making process in the current tasks.

### A beta-band frequency shift signals the categorical decision outcome

Having established a link between prefrontal beta rhythms and decision-making, we proceeded to test our main hypothesis: a shift in beta-band frequency signaled decision outcomes in the human prefrontal cortex. This hypothesis was based on our recent findings in macaques, where distinct beta-band frequencies reflected the categorical decision outcomes in a temporal categorization task.^9^ To this end, we ran an identical version of the temporal categorization task^20^ in humans while recording the EEG. The goal of the current analyses is to test whether this finding in the macaque brain generalizes to the human brain.

**Figure 3:**
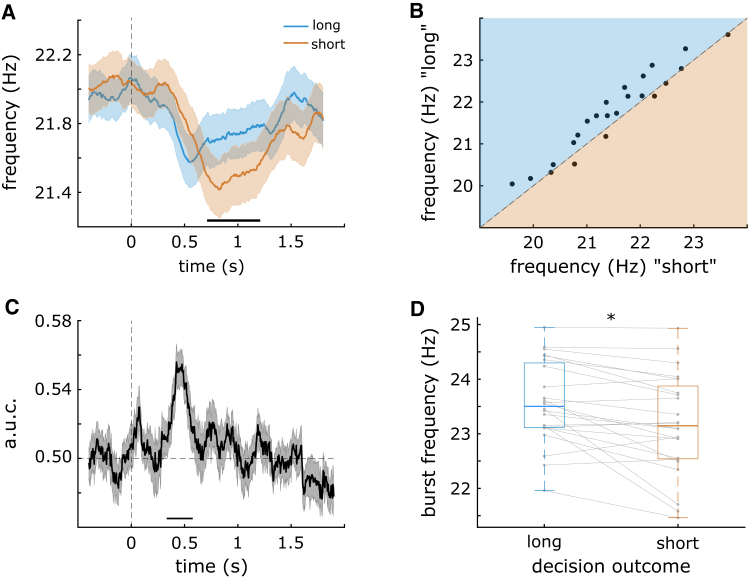
“Long” decisions were associated with a higher beta frequency than “short” decisions (A) Instantaneous frequency in left dlPFC was increased during the decision delay for “long” (blue) vs. “short” decisions (orange). (B) 17/24 participants showed an increase in frequency on “long” vs. “short” trials, while 7 showed a decrease. (C) The decision could be decoded from the instantaneous frequency of sensor-level EEG data. (D) Beta bursts in left dlPFC had a higher peak frequency during the decision delay for “long” vs. “short” decisions. Shaded regions around the line graphs in (A) and (C) represent the standard error of the mean. Horizontal black lines above the *x* axis in (A) and (C) represent the clusters driving the significant differences. Asterix in (D) represents a significant difference (*p* <0 .05).

We found that during the decision delay, a shift in beta-band frequency in the prefrontal cortex signaled the categorical decision outcome (Figure 3). Instantaneous frequency analysis revealed that during the decision delay, prefrontal beta frequency was increased on trials where the time intervals were correctly categorized as “long” compared with “short” (*p*_*cluster*_ = 4e^−4^; Figure 3A). This increase was driven by a cluster containing left vmPFC and left dlPFC (peaking in dlPFC) and ranging from 700 to 1,212 ms after the onset of the decision delay. Within the dlPFC cluster, the average difference in instantaneous frequency was 0.1 Hz, with 17 participants showing a positive difference and 7 participants showing a negative difference (Figure 3B), i.e., more than two-thirds of participants showed a frequency shift in one direction while less than a third showed a difference in the opposite direction. In absolute terms, the average frequency shift within the dlPFC cluster was 0.26 Hz. There was no concurrent difference in spectral beta power between the two decisions (no significant clusters). Compared with the pre-stimulus baseline, instantaneous frequency decreased for both trial types (short: *p*_*cluster*_ = 4e^−4^; long: *p*_*cluster*_ = 4e^−4^), meaning that beta frequency generally decreased from baseline to decision period, but more so on “short” trials. There were no baseline differences in instantaneous frequency between the two trial types.

As a control analysis, we tested whether this pattern of results was reversed on incorrectly categorized trials. We found no significant difference on trials where the time intervals were incorrectly categorized as “long” vs. “short” (no significant clusters). However, including those incorrect trials in the main contrast, such that all subjective “short” vs. “long” decisions were included regardless of whether or not the decision was correct, yielded the same result as including only correct trials (*p*_*cluster*_ = 0.011). To further control to ensure that the differences were driven by stimulus properties rather than decision outcomes *per se*, we compared instantaneous frequency on the same stimulus category but different responses, as well the control grouping of trials with the same response but different stimuli. Among these four contrasts, only that of the short stimulus, short vs. long response was significant (*p*_*cluster*_ = 3e^−4^; long stimulus, short vs. long response: ns; short response, short vs. long stimulus: ns; long response, short vs. long stimulus: ns). While we cannot draw strong conclusions from these control analyses as they relied on sub-groupings with fairly low trial numbers, at the very least they align with a difference based on decision outcome, not stimulus properties.

Next, we used multivariate pattern analysis to ask whether the instantaneous frequency could predict categorical decisions based on the sensor-level EEG data. This analysis has the additional benefit that it is agnostic to the direction of any frequency shifts. We found that “long” vs. “short” decisions could be decoded from beta-band instantaneous frequency during the decision delay (peak area under curve = 0.56, *p*_*cluster*_ = 2e^−4^; Figure 3C). The significant time-resolved decoding was driven by a cluster ranging from 380 to 566 ms after the offset of the stimulus.

Finally, since beta oscillations are often characterized as transient “bursts” of high-amplitude activity,^22^^,^^23^ we asked whether beta burst frequency also signaled the categorical decision. We zoomed in on the left dlPFC, the brain region that displayed the greatest increase in instantaneous frequency for “long” vs. “short” trials. Consistent with the aforementioned findings, we found that during the decision delay, dlPFC beta burst frequency was increased on trials correctly categorized as “long” vs. “short” (t[23] = 3.7, *p* = 0.001; Figure 3D). The burst frequency difference was on average at 0.39 Hz, with 18 participants showing an increase in burst frequency for “long” vs. “short,” and 6 participants showing a shift in the opposite direction. In absolute terms, the average burst frequency shift in dlPFC was 0.45 Hz. The mean burst rate was 2.87 on “long” trials (SD 0.38, *t*-test vs. zero: t[23] = 36.7, *p* < e−12) and 3.00 on “short” trials (SD 0.30, *t*-test vs. zero: t[23] = 48.9, *p* < e−12).

In summary, based on multiple analyses, we find converging evidence that a beta-band frequency shift in the prefrontal cortex signals categorical decisions in this time categorization task.

### The link between beta-band frequency shifts and decisions generalizes to different tasks

Having shown that the categorical decision signal we had initially found in macaque local field potentials generalized to human EEG, we next asked whether it also generalized to other tasks requiring different types of decisions. Furthermore, we asked whether the signal could be detected using a different recording technique. To answer this, we analyzed data from two MEG experiments: a delayed match-to-sample task where the decision was either a “match” or “mismatch” between two previously presented stimuli, and a cross-modal discrimination task where the decision was whether a rhythmic stimulus was “faster” or “slower” than a previously presented rhythmic stimulus.

**Figure 4:**
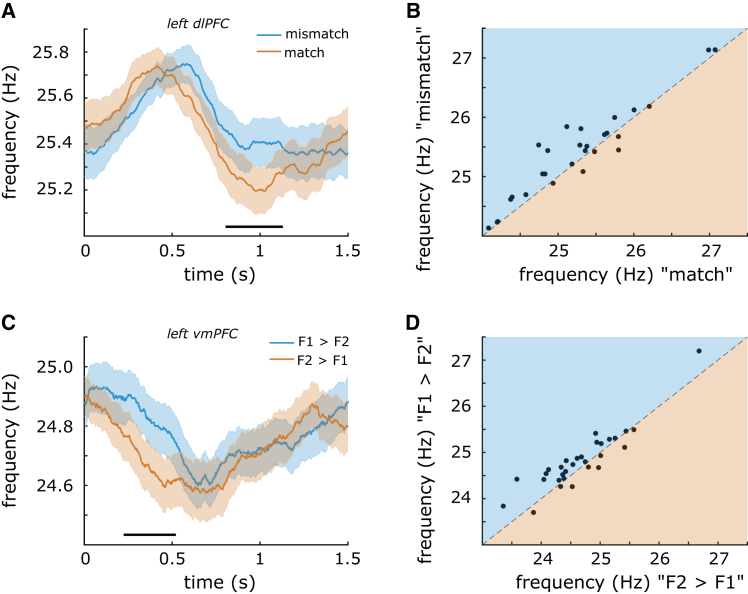
A beta-band frequency shift signaled decision outcomes in two different MEG experiments (A) Instantaneous frequency in left dlPFC was increased during the decision delay for “mismatch” (blue) vs. “match” decisions (orange). (B) 22/28 participants showed an increase in frequency on “mismatch” vs. “match” trials, while 6 showed a decrease. (C) Instantaneous frequency in left vmPFC was increased during the decision delay for “F1>F2” (blue) vs. “F2>F1” (orange) decisions. (D) 22/30 participants showed an increase in frequency on “F1>F2” vs. “F1>F2” trials, while 8 showed a decrease. Shaded regions around the line graphs in (A) and (C) represent the standard error of the mean. Horizontal black lines above the *x* axis in (A) and (C) represent the clusters driving the significant differences.

In the delayed match-to-sample experiment, we found that a beta-band frequency shift in the prefrontal cortex signaled the yes/no decision outcome. Instantaneous frequency analysis revealed increased beta frequency on trials where participants correctly decided the stimuli were a “mismatch” vs. “match” (*p*_*cluster*_ = 0.025; Figure 4A). The increase was driven by a cluster including left dlPFC and vmPFC (peaking in dlPFC), ranging from 790 to 1,110 ms after the onset of the decision delay. Within the left dlPFC cluster, the average difference in instantaneous frequency was 0.14 Hz, with 22 participants showing a positive difference and 6 participants showing a negative difference (Figure 4B). In absolute terms, the average frequency shift was 0.22 Hz. There was no concurrent difference in spectral beta power between the two decisions (no significant clusters). Compared to the pre-stimulus baseline, instantaneous frequency increased for both trial types (match: *p*_*cluster*_ = 0.007; mismatch: *p*_*cluster*_ = 0.009), meaning that beta frequency generally increased from the baseline to the decision period, but more so on “mismatch” trials. There were no baseline differences in instantaneous frequency between the two trial types.

On incorrectly performed trials, there was no significant difference between “match” and “mismatch” decisions (no significant clusters). When including those incorrect trials in the main contrast, such that all subjective “mismatch” vs. “match” decisions were included regardless of whether or not the decision was correct, we found the same result as including only correct trials (*p*_*cluster*_ = 0.004). Further sub-grouping the trials into those having the same stimulus category and contrasting the different responses within those groups did not yield any significant result, and neither did the control grouping of trials with same responses but different stimuli. Next, we used multivariate pattern analysis to ask whether the instantaneous frequency could predict “match” vs. “mismatch” decisions based on the sensor-level data, but the decoder did not perform above chance-level (no significant clusters). Finally, despite a numerical difference in the expected direction, there was no significant difference between the spectral frequency of bursts on “mismatch” vs. “match” trials (t[*27*] = 1.7, *p* = 0.10). The mean burst rate during the decision delay was 2.17 on “mismatch” trials (SD 0.27, *t*-test vs. zero: t[*27*] = 41.2, *p* < e−12) and 2.23 on “match” trials (SD 0.29, *t*-test vs. zero: t[*27*] = 41.9, *p* < e−12).

In the cross-modal discrimination task, we found that a beta-band frequency shift in the prefrontal cortex signaled the faster/slower decision outcome. Instantaneous frequency analysis revealed increased beta frequency on trials where participants correctly discriminated the stimuli as being slower than the previously presented one (*p*_*cluster*_ = 0.022; Figure 4C). The increase was driven by a cluster including left dlPFC and vmPFC (peaking in vmPFC), ranging from 240 to 520 ms after the onset of the decision delay. Within the left vmPFC cluster, the average difference in instantaneous frequency was 0.16 Hz, with 22 participants showing a positive difference and 8 participants showing a negative difference (Figure 4D). Consistent with the results from the other two experiments, more than two-thirds of participants showed a frequency shift in one direction, while less than a third showed a shift in the opposite direction. In absolute terms, the average frequency shift within the vmPFC cluster was 0.24 Hz. Concurrently, spectral beta power was increased on “slower” vs. “faster” trials (*p*_*cluster*_ = 0.041). The increase was driven by a cluster in the left vmPFC ranging from 20 Hz to 24 Hz. Compared to the pre-stimulus baseline, instantaneous frequency increased for both trial types (F2>F1: *p*_*cluster*_ = 0.008; F1>F2: *p*_*cluster*_ = 0.006), meaning that beta frequency generally increased from the baseline to decision period, but more so on “slower” trials. There were no baseline differences in instantaneous frequency between the two trial types.

On incorrectly performed trials, there was no significant difference between “faster” and “slower” decisions (no significant clusters). When including those incorrect trials in the main contrast, such that all subjective “faster” vs. “slower” decisions were included regardless of whether or not the decision was correct, we found the same result as when we had included only correct trials (*p*_*cluster*_ = 0.001). Further sub-grouping the trials into those having the same stimulus category and contrasting the different responses within those groups did not yield any significant results, and neither did the control grouping of trials with same responses but different stimuli. Next, we used a multivariate pattern analysis to ask whether the instantaneous frequency could predict “slower” vs. “faster” decisions based on the sensor-level data, but the decoder did not perform above chance-level (no significant clusters). Finally, despite a numerical difference in the expected direction, there was no significant difference between the spectral frequency of bursts on “slower” vs. “faster” trials (t[29] = 1.8, *p* = 0.08). The mean burst rate during the decision delay was 2.42 on “slower” trials (SD 0.32, *t*-test vs. zero: t[29] = 42.3, *p* < e−12) and 2.31 on “faster” trials (SD 0.28, *t*-test vs. zero: t[29] = 44.6, *p* < e−12).

### The frequency shift is not restricted to the prefrontal cortex

**Figure 5:**
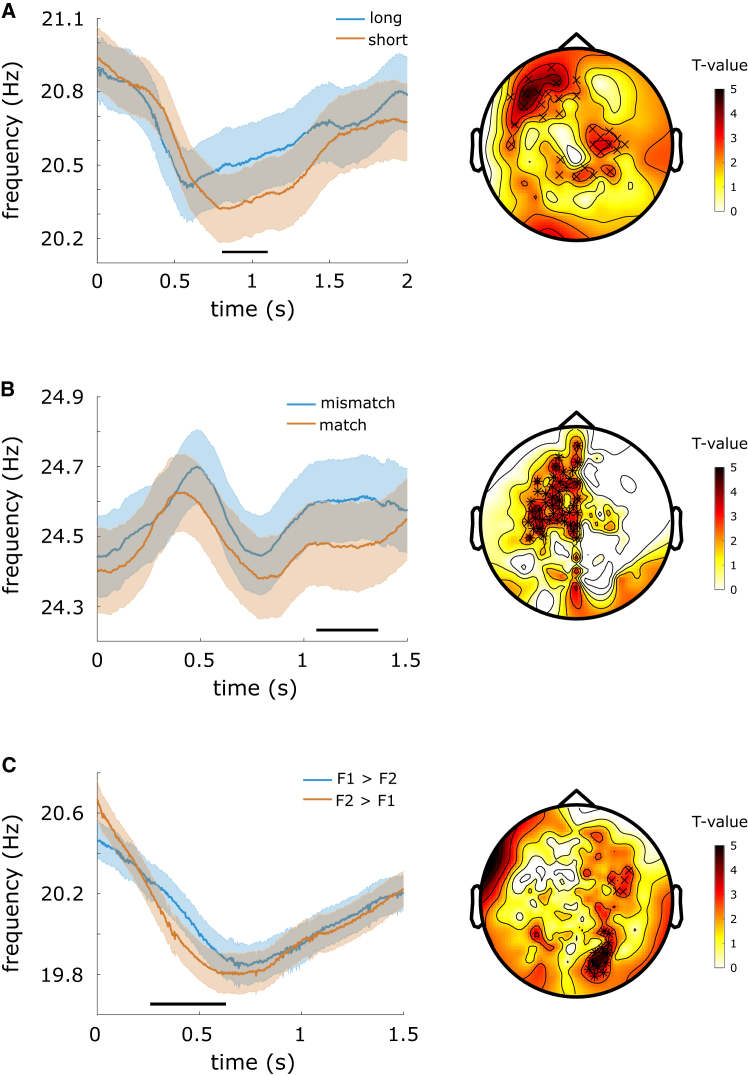
The beta-band frequency shift was observed on sensor level beyond frontal sensors (A) Instantaneous frequency (left) in the highlighted EEG channels (right) was increased during the decision delay for “long” (blue) vs. “short” decisions (orange). (B) Instantaneous frequency (left) in the highlighted MEG channels (right) was increased during the decision delay for “mismatch” (blue) vs. “match” decisions (orange). (C) Instantaneous frequency (left) in the highlighted MEG channels (right) was increased during the decision delay for “F1>F2” (blue) vs. “F2>F1” decisions (orange). Shaded regions around the line graphs in the left panels represent the standard error of the mean. Horizontal black lines above the *x* axis in the left panels represent the clusters driving the significant differences. The topographies in the right panels represent the average *t*-values (per sensor) within those clusters driving the significant differences. The sensors contributing to the clusters are marked (^x^*p* < 0.05, ∗*p* < 0.01).

After showing that the categorical signal generalized across EEG and MEG experiments using our region-of-interest approach in source space, we additionally explored the spread of these effects on the sensor level. In the case of all three experiments, we found that a beta-band frequency shift in the sensor data signaled the decision outcome. The effects were qualitatively and quantitatively similar to those we observed in the source data (Figure 5).

In the temporal categorization task, beta frequency during the decision delay was increased on trials where the time intervals were correctly categorized as “long” compared to “short” (p_cluster_ = 0.013; Figure 5A left). The increase was driven by a cluster of EEG sensors including left frontal sensors and central sensors (Figure 5A right), ranging from 774 to 1,144 ms after the onset of the decision delay. In the delayed match-to-sample task, beta frequency during the decision delay was increased on trials where the participants correctly decided the stimuli were a “mismatch” vs. “match” (*p*_*cluster*_ = 0.003; Figure 5B left). The increase was driven by a cluster of MEG sensors including left frontal and central sensors (Figure 5B right), ranging from 850 to 1,217 ms after the onset of the decision delay. In the cross-modal discrimination task, beta frequency during the decision delay was increased on trials where the participants correctly discriminated the stimulus as being slower than the previously presented one (*p*_*cluster*_ = 0.007; Figure 5C left). The increase was driven by a cluster of MEG sensors including left frontal sensors and right posterior and central sensors (Figure 5C right), ranging from 850 to 1,217 ms after the onset of the decision delay.

In summary, the instantaneous frequency time-courses and differences in sensor space largely matched those we observed in source space. While primarily centered on frontal sensors, the spread of the effects in sensor space also included posterior and central sensors.

## Discussion

Our results show that beta-band frequency shifts are a decision signal in the human prefrontal cortex and likely other brain areas, robust across task designs, decision types, stimulus modalities, analysis approaches, and recording techniques. In three separate EEG and MEG experiments (*n* = 82), we used tasks that required participants to accurately decide between two alternative choices. The task designs were such that the decision was dissociated from the subsequent motor response, precluding any preparatory motor effects. Using both a source-level ROI approach and a whole-brain sensor-level approach, we found that the frequency shift consistently signaled the decision outcome, despite the tasks employing different kinds of stimuli and requiring different types of decisions. Having first observed this signal in macaques performing a temporal categorization task,^9^ we here show that this finding translates to humans performing the same task as well as different tasks. Note that while our experimental design operationalized decision-making from initial stimulus to motor response as a serial process (as the correspondence between decision and action is not known before the response mapping is shown), that was merely to avoid both sensory and motor confounds in the analysis of the decision window. We do not mean to imply that the neural processes involved are strictly serial in nature. Likely, the beta frequency shifts we observe in the prefrontal cortex are one aspect of continuous dynamics spanning a distributed network, reflecting an integrated process from stimulus perception to motor execution of the decision outcome.

In each of the three experiments, the decision outcome was significantly mapped onto beta frequency—meaning a slower frequency was associated with one decision and a faster frequency associated with another decision—though the direction (or sign) of the effect differed across participants. Interestingly, in each experiment the direction of the shift was the same in more than two-thirds of participants (70%–78%). In the temporal categorization task, results from macaques showed that a slower frequency was associated with the “long” category while a faster frequency was associated with the “short” category, and this result was consistent across the two tested macaques. Based on this result, it would have been tempting to hypothesize that slower frequencies are associated with decisions about stimuli with relatively larger magnitudes. However, in the current EEG temporal categorization task, the mapping between frequency and relative stimulus category was reversed in most participants compared to the macaques. Further, the two MEG experiments involved decisions about comparisons between two sequentially presented stimuli, with the delayed match-to-sample task not involving any category or magnitude judgements at all. Despite this, we observed different frequencies consistently mapping to specific decisions, with about two-third of participants showing one pattern and one-third the opposite. While we do not have any conclusive evidence as to whether beta frequencies map onto decisions in a principled or arbitrary way, we do not see any grounds to suggest that a decision will map onto the same frequency consistently across individuals. Instead, we hypothesize that a flexible mapping comes about in response to learning and rule updating. For example, we hypothesize that adding more possible decision outcomes would result in additional distinct beta-frequency channels that can be flexibly shifted (i.e., adding more categories or updating rules would also shift the frequencies of the original categories). These hypotheses could be addressed in follow-up experiments.

The frequency shifts observed in the EEG categorization experiment were highly robust, more so than those observed in the MEG experiments. For example, we found that single-trial responses could be successfully decoded from the instantaneous frequency signal in the EEG data but not from the MEG data. Similarly, we found that bursts in the beta range shifted in frequency according to the decision outcomes in the EEG experiment, while in the MEG experiments the burst analysis showed only non-significant trends in the expected directions. One reason for this could be that the EEG categorization experiment is a direct replication of the categorization experiment in macaques where the effect was originally reported, while the MEG experiments were completely different tasks, and that perhaps there is something specific about temporal categorization that elicits these (stronger) beta shifts. For example, it is possible that the temporal categorization task is more difficult than the other tasks and effects are more visible when cognitive demands are higher. Another reason might be that EEG is more sensitive to frontal activity than MEG, since participants typically rest their heads toward the back of the MEG helmet, placing frontal brain areas farther away from the MEG sensors and thereby rendering MEG potentially less sensitive to frontal activity. Therefore, a limitation of this study is that we cannot conclusively disambiguate these possibilities based on the current datasets, as the lack of significant burst findings could be explained either by the differences in methods (EEG vs. MEG) or by the differences in tasks.

To further support our finding that the frequency shift represented participants’ subjective decisions and not objective stimulus properties, we repeated our analysis on the incorrectly performed trials, expecting a reversal in the direction of the frequency shift. This control analysis did not yield any significant result, likely due to the low number of incorrect trials (around 20%, on average 30 trials per condition). However, including the incorrect decisions as part of the main contrast (that is, all A decisions whether correct or incorrect vs. all B decisions whether correct or incorrect) did yield significant effects similar to those we found when only including correct trials. To thoroughly investigate a possible reversal of the effect on incorrect trials, thereby replicating our previous finding in macaques,^9^ future experiments should titrate task difficulty such that there are sufficient incorrect trials to analyze. As an additional control, we tested for concurrent changes in spectral beta power between the two decisions, as such power modulations have been previously reported in decision-making experiments; e.g.,^24^^,^^25^^,^^26^^,^^27^^,^^28^ we only found a concurrent power difference in the cross-modal discrimination MEG experiment, but not in the other experiments. One main difference between our experiment and those of Donner and colleagues^24^^,^^28^ is that our design has a built-in control for motor preparatory effects which also manifest as beta power modulations. Our cross-modal discrimination MEG experiment is similar to that of Herding and colleagues,^25^^,^^26^ so it is possible that beta power differences appear in such settings but not others. Although differences in frequency and differences in power are theoretically independent, it has been shown that frequency differences can lead to observed power differences.^16^ We conclude that a frequency shift is a more robust marker of decisions compared to a change in power.

Additionally, we observed a decrease in beta power (as well as a frequency shift) during the decision compared to the baseline window, a finding which can be interpreted within the classical view as reflecting changes in inhibitory states. One possibility is that changes in power and frequency are independent phenomena, and that a frequency shift can occur despite there being less power. Another possibility is that during rest or an inhibitory state (high beta power), all frequency channels within the beta band are inhibited, while during decision-making, one of the frequency channels is disinhibited by a top-down mechanism, resulting in an overall decrease in beta power and a shift in frequency. The shift in frequency from baseline to decision delay varied in each experiment, such that it decreased in the EEG experiment but increased in the MEG experiments, but we again cannot disentangle whether this is a task- or method-related difference. Although much work has highlighted the potential role of beta in top-down control of sensorimotor and cognitive states (for a review, see Spitzer and Haegens, 2017), there is also a growing body of evidence suggesting that beta bursts are involved in bottom-up reactive control^4^^,^^29^ or sensory processing.^30^ For example, Wessel and colleagues found that beta bursts were involved in both movement initiation and reactive motor inhibition^4^ or surprise.^29^ Beta dynamics are likely multifaceted phenomena, and changes in power, frequency, bursting properties, cortical focus, and connectivity profile could reflect different cognitive and/or sensorimotor processes.

A biophysically plausible account of beta oscillations in cortex posits that they emerge due to synchronized bursts of subthreshold excitatory synaptic input, simultaneously activating both proximal and distal dendrites of pyramidal neurons.^31^^,^^32^^,^^33^ Within this model, the duration of the distal drive is inversely related to beta frequency, providing a biophysical mechanism that leads to a frequency shift. Since the beta-band oscillatory dynamics in the model are subthreshold, our reasoning is that these dynamics then impact (or modulate) spiking activity in these pyramidal populations, and thereby the subsequent downstream (interareal) communication. For example, despite the generated current being subthreshold itself, it could influence the timing of spikes from these same populations, thereby contributing to a frequency code at the population level. Modeling work has shown that frequency-division multiplexing (or frequency coding) is an efficient mechanism by which oscillations at particular frequencies can transmit population codes to networks which can then read the code given appropriate filter settings.^34^^,^^35^ A frequency shift could therefore serve to selectively transmit task-relevant information (e.g., a decision) downstream through a dedicated frequency channel.

Although frequency shifts within the beta band are a novel observation, shifts within the alpha and theta bands have been reported and linked to visual processes^36^^,^^37^^,^^38^^,^^39^ and cognitive control,^40^^,^^41^ respectively. We here propose a new role for beta frequency shifts in decision-making. We previously proposed that beta rhythms serve to activate and/or reactivate neuronal ensembles that encode endogenous content, and that changes in beta power mark this reactivation process.^11^ However, changes in frequency can often co-occur with changes in power, so investigating power exclusively does not reveal the full picture.^16^ Based on the current set of results, we now extend our framework and propose that distinct beta frequencies map onto, and provide read-outs of, endogenous contents (in this case, decisions). As such, they can be viewed as biomarkers of decision-making.

### Limitations of the study

There were some differences between the results of the three experiments, in particular the robustness of the burst and decoding results. These differences could be due to the different tasks used (perhaps some tasks are more difficult than others) or the different recording methodologies (EEG vs. MEG). A main limitation of this study is that we cannot conclusively disambiguate these possibilities.

## Resource availability

### Lead contact

Requests for further information and resources should be directed to and will be fulfilled by the lead contact, Elie Rassi (elie.r.rassi@gmail.com).

### Materials availability

This study did not generate new unique reagents.

### Data and code availability

• The data are openly available here https://osf.io/a5m7q/.
• The code is also openly available here https://osf.io/a5m7q/, as listed in the key resources table.
• Any additional information required to reanalyze the data reported in this paper is available from the lead contact upon request.

## Acknowledgments

E.R. is supported by the Austrian Science Fund (FWF), Erwin Schrödinger Fellowship (J4580), and NWO OCENW.XL21.XL21.069 (SoMeMe). H.M. is supported by Consejo Nacional de Humanidades, Ciencia y Tecnología (CONAHCYT) (CBF-2025-G-89), and UNAM-DGAPA-PAPIIT (IG200424). S.H. is supported by 10.13039/501100003246NWO Vidi 016.Vidi.185.137, 10.13039/100000002NIH R01 MH123679, and NSF CRCNS 2424100. The authors would like to thank Anna Aumeistere for help with data collection.

## Author contributions

E.R., J.R.L., A.E., H.M., and S.H. designed the research. E.R., J.R.L., C.G., and A.E. collected the data. E.R., J.R.L., C.G., and A.E. helped process and analyze the data. E.R. analyzed the data and wrote the manuscript. E.R., J.R.L., H.M., and S.H. edited the manuscript.

## Declaration of interests

The authors declare no competing interests.

## STAR★Methods

### Key resources table

**Table:** 

REAGENT or RESOURCE	SOURCE	IDENTIFIER
**Deposited data**		

Experimental data (EEG & MEG)	Elie El Rassi	https://doi.org/10.17605/OSF.IO/A5M7Q

**Software and algorithms**		

Code	Elie El Rassi	https://doi.org/10.17605/OSF.IO/A5M7Q

### Experimental model and study participant details

Experiment 1 *(temporal categorisation)*: 27 healthy adults (15 female; mean age 25.6 +/- 4.2 years) participated in the temporal categorisation experiment. They all reported normal or corrected-to-normal vision and no history of neurological or psychiatric disorders. The study was approved by the Institutional Review Board (IRB) of the New York State Psychiatric Institute (protocol # 8001). Participants gave written informed consent and were compensated for their participation (at 25 USD per hour). Three participants were excluded from the analysis due to technical problems during data acquisition.

Experiment 2 *(delayed match-to-sample)*: 28 healthy adults (15 female; mean age 26.7 +/- 3.6 years) participated in the delayed match-to-sample experiment. They all reported normal or corrected-to-normal vision and no history of neurological or psychiatric disorders. The study was approved by the local Arnhem-Nijmegen ethics committee (CMO 2014/288 “Imaging Human Cognition”). Participants gave written informed consent and were compensated for their participation (at 10 EUR per hour).

Experiment 3 *(cross-modal discrimination)*: 32 healthy adults (17 female; mean age 25.9 +/- 5.4 years) participated in the cross-modal discrimination experiment. They all reported normal hearing, normal or corrected-to-normal vision, and no history of neurological or psychiatric disorders. The study was approved by the local Arnhem-Nijmegen ethics committee (CMO 2014/288 “Imaging Human Cognition”). Participants gave written informed consent and were compensated for their participation (at 10 EUR per hour). Two participants were excluded from the analysis due to technical problems during data acquisition.

### Method details

All three experiments were programmed with PsychtoolBox^42^ in Matlab (Mathworks, Inc.). Each of the experiments had a decision-making component, where participants were forced to choose between two alternatives (with each alternative being correct 50% of the time), after a fixed decision delay. A response map, randomised on a trial-by-trial basis, appeared after the decision delay, prompting participants to respond by pressing one of two buttons (Figure 1A).

Experiment 1 (*temporal categorisation;* Figures 1A and 1C): Task and stimuli were based on our previous studies investigating categorical decision-making in macaques.^9^^,^^20^^,^^21^ Participants performed a temporal interval categorization task, where they had to categorise a visual stimulus as “long” or “short” based on its duration on screen. Each trial started with the presentation of a fixation cross for 2 s. Then, the to-be-categorized stimulus (a circle around the fixation cross) would be shown for a specific time interval. After another 2-s delay (the decision delay), participants had to report whether the presented stimulus was “short” or “long” by pressing the right or left arrow key on a computer keyboard. To avoid motor preparation confounds in our decision-related analyses, we randomly changed response mappings (i.e., left vs. right arrow key) on a trial-by-trial basis. Feedback was presented at the end of each trial via a coloured fixation cross (green for correct and red for incorrect responses). The inter-trial interval (ITI) was 500 ms.

The task involved three stimulus sets (T1, T2 and T3) with different interval durations. There were eight interval durations per set, four of which short and four long. The two longest intervals in T1 were equal in magnitude to the two shortest intervals in T2, and the two longest intervals in T2 were equal in magnitude to the two shortest intervals in T3. That is, we shifted the categorical boundary between sets, such that the same stimulus could be considered short in one set but long in another. The interval durations in T1 were 200, 250, 319, 331, 369, 381, 450, and 500 ms. The interval durations in T2 were 450, 500, 619, 669, 706, 756, 870, and 920 ms. The interval durations in T3 were 870, 920, 981, 1169, 1231, 1419, 1470, and 1520 ms. These were the exact same stimulus sets as used in the macaque work.^20^

This task design allowed us to probe decisions about relative categorical decisions rather than decisions about objective stimulus properties. We presented the different sets in a blocked design, with the order of blocks randomised per participant. Each block had a learning phase of 10 trials where we presented the longest and shortest durations of that set, allowing participants to learn the meaning of “short” and “long” durations for that set and implicitly learn the categorical boundary. We recorded EEG while participants performed a total of 336 trials (112 per block), with the experiment lasting approximately 1 hour. More information on this task, as well as additional findings from this dataset can be found in our prior work.^43^

Experiment 2 (*delayed match-to-sample;* Figures 1A and 1D): Task and stimuli were based on our previous study investigating the role of neural oscillations in working memory.^44^ We used a delayed match-to-sample working-memory task where participants were instructed to compare one of two samples with a probe stimulus and indicate whether the cued sample was of the same or different spatial frequency as the probe. Each trial consisted of the following events: a visual cue followed by two visual samples, a second visual cue, a probe and a response mapping diagram. All events, except the probe, were presented at a fixed stimulus onset asynchrony (SOA) of 1.5 s. Per block, the probe was presented either at an early (1.5 s), late (3.5 s) or jittered (1.5–3.5 s) SOA. The response-button mapping changed on a trial-by-trial basis to avoid motor preparation confounds in our decision-related analyses. The ITI was jittered between 2 and 2.4 s.

We used a three (probe SOA: early vs. late vs. jittered) by two (relevant sample: first vs. second) by two (informative cue: pre- vs. retro-cue) factorial design. Participants were cued to compare the probe’s spatial frequency to the first sample in half of the trials and to the second sample in the other half. They were informed about the relevant sample by an informative cue, which occurred either before (pre; 50%) or after (retro; 50%) sample presentation. These four trial types were randomly interleaved such that within each block participants performed all task conditions. Once presented with the response mapping diagram, participants gave their response (“match” or “no match”) by pressing a button with their right index or middle finger.

A bull’s eye was presented at the centre of the screen as the fixation point. Each trial started with a cue presented for 300 ms. Next, the first sample consisting of an oriented grating (Michelson contrast: 40%, spatial frequency: 1 cycle per °, orientation: vertical, randomised spatial phase) was presented for 100 ms. The gratings were shown in an annulus (inner radius = 1.5°, outer radius = 7.5°, contrast of the stimuli decreased linearly to 0 over the outer and inner 0.5° radius of the annulus) around the central fixation. After an SOA of 1.5 s, a second grating was presented, followed by a second cue. Next, the probe was presented, which, similar to the sample, consisted of an oriented grating. The trial presentation finished with the response mapping presentation. The cued sample’s spatial frequency matched that of the probe grating in 50% of the trials.

Participants first performed a few short practice sequences of the task (20 trials each) in which feedback was provided on a trial-by-trial basis, with green and red fixation dots for correct and incorrect responses, respectively. During the main experiment, we recorded MEG while participants performed eight blocks of 32 trials each, in which feedback was provided on a block-by-block basis. Afterwards, participants underwent a block where they passively viewed the gratings presented during the main block. During this localiser block, participants viewed 300 gratings with 1.5-s SOA. The full recording session lasted around 1.5 hour. More information on this task, as well as additional findings from this dataset can be found in our prior work.^45^

Experiment 3 (*cross-modal discrimination;* Figures 1 and 1E): Task and stimuli were based on our previous work investigating the role of beta rhythms in perceptual decision-making in macaques.^27^^,^^46^ Here, we used a cross-modal discrimination task where participants were presented with two sequential vibrating (auditory or tactile) stimuli, and had to indicate whether the second stimulus was faster or slower than the first. Each trial started with a cue lasting 500 ms, after which two stimuli were sequentially presented for 500 ms each, with a 1.5-s interval in between them. After the second stimulus, there was a decision delay of 1.5 s, after which participants had to respond within 2 s. We randomised the response mapping to avoid motor preparation confounds in our decision-related analyses. The ITI was jittered between 1 and 2 s.

Trials were either unimodal (i.e., tactile-tactile or auditory-auditory) or cross-modal (i.e., tactile-auditory or auditory-tactile) and randomly intermixed. These four trial types were either predictable (informative cue, 50% of trials) or unpredictable (uninformative cue, 50% of trials). On predictable trials, a visual symbolic cue indicated whether the trial was unimodal or cross-modal, such that after presentation of the first stimulus, the subject would know in which modality to expect the second stimulus. In all cases, participants had to compare the frequency (or speed) of the two stimuli and indicate whether the second was faster or slower than the first, regardless of their modality.

The tactile stimuli were bimanual vibro-tactile (electrical stimuli via two constant-current high-voltage stimulators; Digitimer Ltd, Model DS7A), and the auditory stimuli were binaural flutter stimuli (auditory pulse trains). The stimuli, lasting 500 ms, could have any of the following frequencies: 10, 12, 16, 20, 22, 24, 28, or 34 Hz. The combinations were pseudo-randomised such that the differences between them would be 2, 4, or 6 Hz.

Participants first performed practice blocks of 20 trials each, and the intensity of the stimuli were adjusted after each practice block such that they felt subjectively equal. When participants’ performance reached 70% on the training blocks, the experiment proper began. The experiment consisted of eight blocks of 40 trials for a total of 320 trials. The full recording session lasted around 1 hour.

#### Data acquisition

Experiment 1: 96-electrode scalp EEG was collected using the BrainVision actiCAP system (Brain Products GmbH, Munich, Germany) with a sampling rate of 500 Hz. Electrodes were labelled according to the international 10-20 system. The reference electrode during the recording was Cz. Amplification and digitalization of the EEG signal was done through an actiCHamp DC amplifier (Brain Products GmbH, Munich, Germany) linked to BrainVision Recorder software (version 2.1, Brain Products GmbH, Munich, Germany). Vertical (VEOG) and horizontal (HEOG) eye movements were recorded by placing additional bipolar electrodes above and below the left eye (VEOG) and next to the left and right eye (HEOG).

Experiments 2 and 3: whole-head MEG data were recorded at a 1200-Hz sampling rate with a 275-channel CTF MEG system with axial gradiometers (CTF MEG Systems, VSM MedTech Ltd.) in a magnetically shielded room. To monitor the participants’ head movements online and for offline co-registration of anatomic landmarks, three fiducial coils were placed at the nasion and both ear canals. Anatomical T1-weighted MRI scans for source localization purposes were obtained in a separate session, using either a 1.5 or 3 T Siemens MRI system (Siemens). To co-register the MEG and MRI data, we additionally mapped the scalp with Polhemus 3D (Polhemus).

#### EEG, MEG, and MRI preprocessing

Experiment 1: EEG data were preprocessed using custom scripts and functions from EEGLAB^47^ and Fieldtrip^48^ toolboxes. Data were first resampled to 250 Hz and filtered between 0.5 and 40 Hz. Noisy electrodes were automatically detected (EEGLAB function clean_channels) and interpolated. EEG data were re-referenced to the common average and independent component analysis (runica algorithm) was performed. An automatic component rejection algorithm (IClabel) was employed to discard components associated with muscle activity, eye movements, heart activity or channel noise (threshold = 0.8; see^49^). In addition, components with an absolute correlation with HEOG, VEOG or ECG channels higher than 0.8 were discarded. Furthermore, Artifact Subspace Reconstruction (ASR) was employed to correct for abrupt noise with a cutoff value of 20 SD. Finally, the data were locked to the onset of the decision delay and segmented (-0.5 to 2 s).

Experiment 2: MEG data were preprocessed offline and analysed using the FieldTrip toolbox^48^ and custom MATLAB scripts. The MEG signal was epoched based on the onset of the first cue (-2 to 7 s). The data were downsampled to a sampling frequency of 300 Hz, after applying a notch filter to remove line noise and harmonics (50, 100, and 150 Hz). Bad channels and trials were rejected via visual inspection before independent component analysis^50^ was applied. Subsequently, components representing eye-related and heart-related artefacts were projected out of the data. Finally, outlier trials of extreme variance were removed. We co-registered the MRI to the CTF coordinate system using the fiducial points and the mapped scalp surface, and segmented the MRI image with SPM8 (as implemented in Fieldtrip).

Experiment 3: MEG data were preprocessed offline and analysed using the FieldTrip toolbox^48^ and custom MATLAB scripts. First, we downsampled the data to a sampling frequency of 400 Hz. Next, we segmented trials into 8-s segments starting 1 s before cue onset. We rejected bad channels (∼5%) and bad trials (∼10%) via visual inspection before independent component analysis (runica as implemented in Fieldtrip), which was used to visually detect and remove components representing eye blinks and heartbeats. We co-registered the MRI to the CTF coordinate system using the fiducial points and the mapped scalp surface, and segmented the MRI image with SPM8 (as implemented in Fieldtrip).

#### EEG and MEG source reconstruction

For the EEG source reconstruction, no MRI scans were available, so we used a standard Boundary Element Method volume conduction model^51^ for all participants. For the MEG source reconstruction, we generated anatomically realistic single-shell headmodels^52^ based on each individual’s T1-weighted anatomical scan. We then created grids with 1-cm resolution using an MNI template onto which the brain volume of each participant was morphed using non-linear transformation. Next, we computed leadfields for the grid points that corresponded to our four pre-defined regions of interest: left dlPFC, right dlPFC, left vmPFC, and right vmPFC (Figure 1B). The MNI coordinates were, respectively: [-32 43 23]; [32 43 23]; [-6 45 -9]; [6 45 -9] mm. We used a linearly constrained minimum variance (LCMV) beamformer approach^53^ to compute the spatial filters at these locations and extract the time-series signal for these four virtual channels.

### Quantification and statistical analysis

#### Spectral power analysis

For the spectral power contrasts (fractal vs. spectral, decision delay vs. baseline), we first segmented the source-space trial data into separate decision and baseline windows of equal length. In Experiment 1, we segmented the 2-s decision window and 2-s baseline window. In Experiment 2, where the decision window was 1.5 s long, we segmented the 1.5-s decision window and 1.5-s baseline window. In Experiment 3, the decision window was also 1.5-s long, but the baseline window was 1-s long at minimum, so here we segmented 1 s from the latter part of the decision window and the 1-s baseline window.

To check whether beta power was significantly elevated above what would be expected from 1/f background, we used IRASA^54^ with default parameters to estimate both the fractal and oscillatory components of the power spectra during the baseline and decision delays. We used a cluster-based permutation approach^55^ to contrast group-level fractal vs. oscillatory spectral power in the beta frequency range (13 to 35 Hz), clustering over frequencies and neighbouring sources (sources in the same hemisphere were considered neighbours, meaning each source had one neighbour).

For the decision delay vs. baseline contrast, we zero-padded the segmented source-level data to 4-s length and used the Fast Fourier Transform as implemented in Fieldtrip, with multitapers (discrete prolate spheroidal sequence) and +/-2 Hz smoothing, thus obtaining spectral power estimates for the frequencies between 8 and 38 Hz. We then used the same cluster-based permutation approach described in the previous paragraph.

#### Instantaneous frequency analysis

To investigate the time course of the peak beta frequency during the decision delays of all three experiments, we analysed instantaneous frequency as detailed by Cohen.^56^ Briefly, we band-passed the single-trial data within the beta frequency range, applied the Hilbert transform, extracted the phase angle time series, took the temporal derivative, and applied ten median filters. This resulted in single-trial estimates of instantaneous frequency during the decision delay for each of the four sources. For the sensor-level analysis, we used the same procedure on data from all EEG and MEG sensors.

To contrast the instantaneous frequency estimates between the two decision outcomes of each experiment, we used a cluster-based permutation approach,^55^ clustering over time points and neighbouring sources (in source space, one neighbour per source) or neighbouring sensors (in sensor space, 6 neighbours per sensor on average). For each experiment, we extracted the individual instantaneous frequency values within the most prominent source-level cluster resulting from the cluster-based permutation statistics. We averaged those (across time points) separately for each participant and each decision outcome to obtain the individual-level frequencies associated with the decision outcomes. To obtain topographies of the effects on the sensor level, we extracted the t-values within the most prominent sensor-level cluster resulting from the cluster-based permutation statistics, averaged those (across time points), and highlighted the sensors contributing to that cluster.

#### Multivariate pattern analysis (decoding)

To investigate whether the instantaneous frequency signalling of the decision outcome was also visible in the sensor-level data, we performed multivariate pattern analysis (MVPA). We used the MVPA-light toolbox^57^ as implemented in Fieldtrip. We defined sensors as features and applied a Linear Discriminant Analysis classifier with 8-fold cross-validation repeated twice. We used Area Under the Curve to quantify the classifier’s performance across time, and a cluster-based permutation approach (clustering across time points) to contrast the classifier’s performance with chance values (0.5).

#### Burst analysis

We detected beta bursts in the source-reconstructed signals during the decision delay with the Spectral Events toolbox,^22^ using the first of three available algorithms (findMethod=1). This algorithm first computes a time-frequency representation of the data (convolution with complex Morlet wavelets, 7 cycles), then finds local maxima, and finally selects suprathreshold peaks within the frequency band of interest and labels them as burst events. We extracted the mean burst rates from these events per subject and condition and contrasted them vs. zero with a one-tailed, paired t-test. We finally extracted the peak frequencies from these events and averaged them per subject and per decision outcome, before contrasting the frequencies representing the two decisions with a two-tailed, paired t-test.
